# Supplementary material for: Rab30 facilitates lipid homeostasis during fasting
Source: Nat Commun. 2024 May 25;15:4469. doi: 10.1038/s41467-024-48959-x (PMC11127972; doi:10.1038/s41467-024-48959-x)
Supplement: Supplementary file 1 — Supplementary Information [file 41467_2024_48959_MOESM1_ESM.pdf]

Supplementary Information

Rab30 facilitates lipid homeostasis during fasting

Danielle M. Smith[1,2], Brian Y. Liu[1], and Michael J. Wolfgang[1,2,3]*

[1]Department of Physiology, The Johns Hopkins University School of Medicine, Baltimore, MD 21205, USA
[2]Biological Chemistry, The Johns Hopkins University School of Medicine, Baltimore, MD 21205, USA
[3] Pharmacology and Molecular Sciences, The Johns Hopkins University School of Medicine, Baltimore, MD 21205, USA

*Address correspondence to**: Michael J. Wolfgang, Ph.D,** Department of Physiology, Johns Hopkins University School of Medicine, 855 N. Wolfe St., 475 Rangos Building, Baltimore, MD 21205, Email: mwolfga1@jhmi.edu

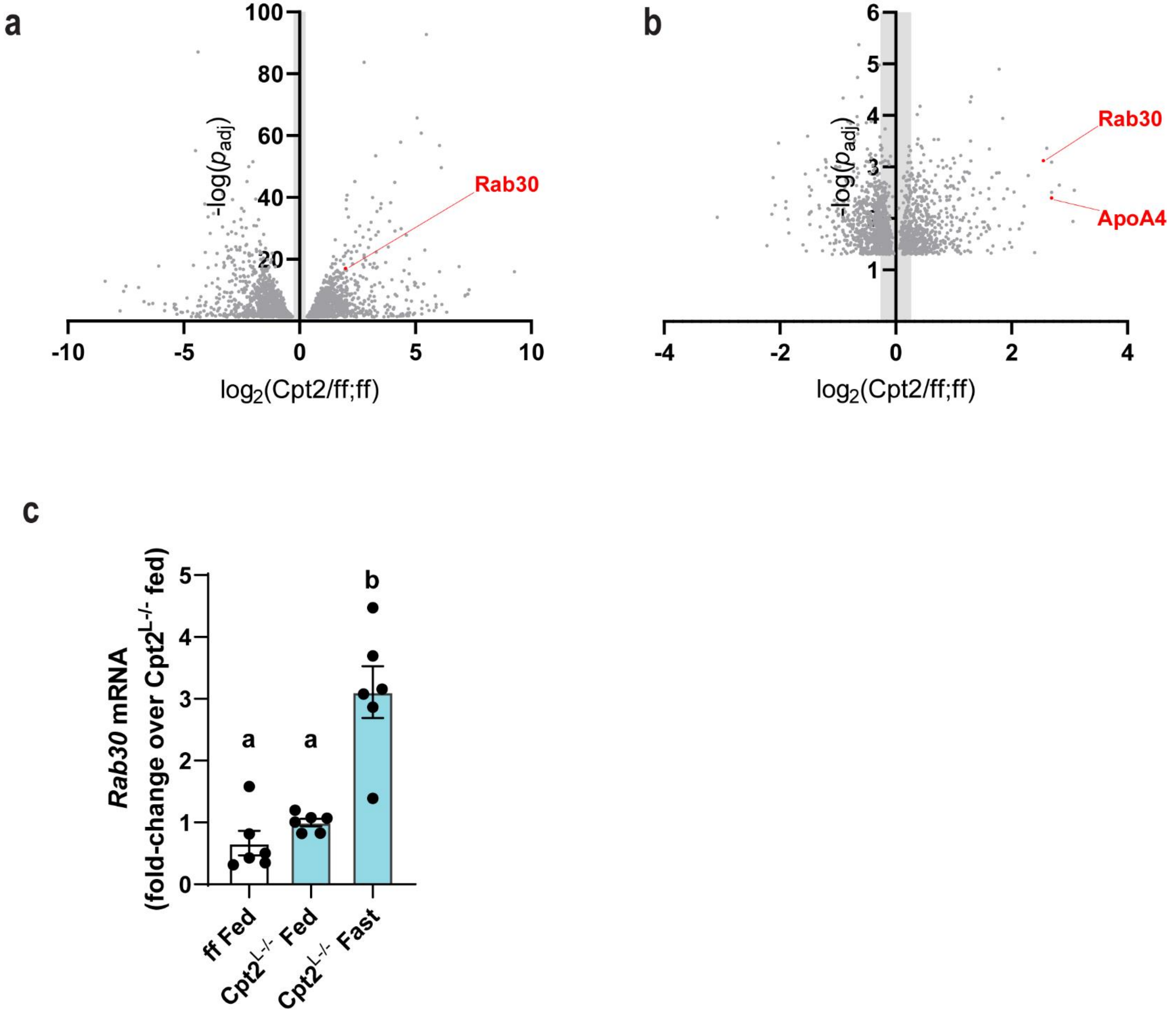
a
-log(p_{adj})
Rab30
$\log_2$(Cpt2/ff;ff)
b
-log(p_{adj})
Rab30
ApoA4
$\log_2$(Cpt2/ff;ff)
c
Rab30 mRNA (fold-change over Cpt2$^{L-/-}$ fed)
a
a
b
ff Fed
Cpt2$^{L-/-}$ Fed
Cpt2$^{L-/-}$ Fast

Supplementary Fig. 1. **Rab30 is amongst the most highly upregulated transcripts and proteins in the fasted livers of mice incapable of β-oxidation. a,b** Volcano plots of differentially expressed (*p*<0.05) (**a**) transcripts from RNA-seq data and (**b**) proteins from proteomics data in 24hr fasted livers of Cpt2$^{L-/-}$ and control (Rab30;Cpt2 double floxed, or ff;ff) animals (n=4/genotype). Gray bars indicate fold-change of less than 1.2. **c** qRT-PCR of hepatic *Rab30* mRNA levels in the fed state of Cpt2 floxed (ff) and Cpt2$^{L-/-}$ males and in the 24hr fasted state of Cpt2$^{L-/-}$ males. Values are mean±SEM relative to Cpt2$^{L-/-}$ fed. Letters indicate significance groups by Tukey's multiple comparisons test following one-way ANOVA. ANOVA tables and source data for relevant panels are provided as a Source Data file.

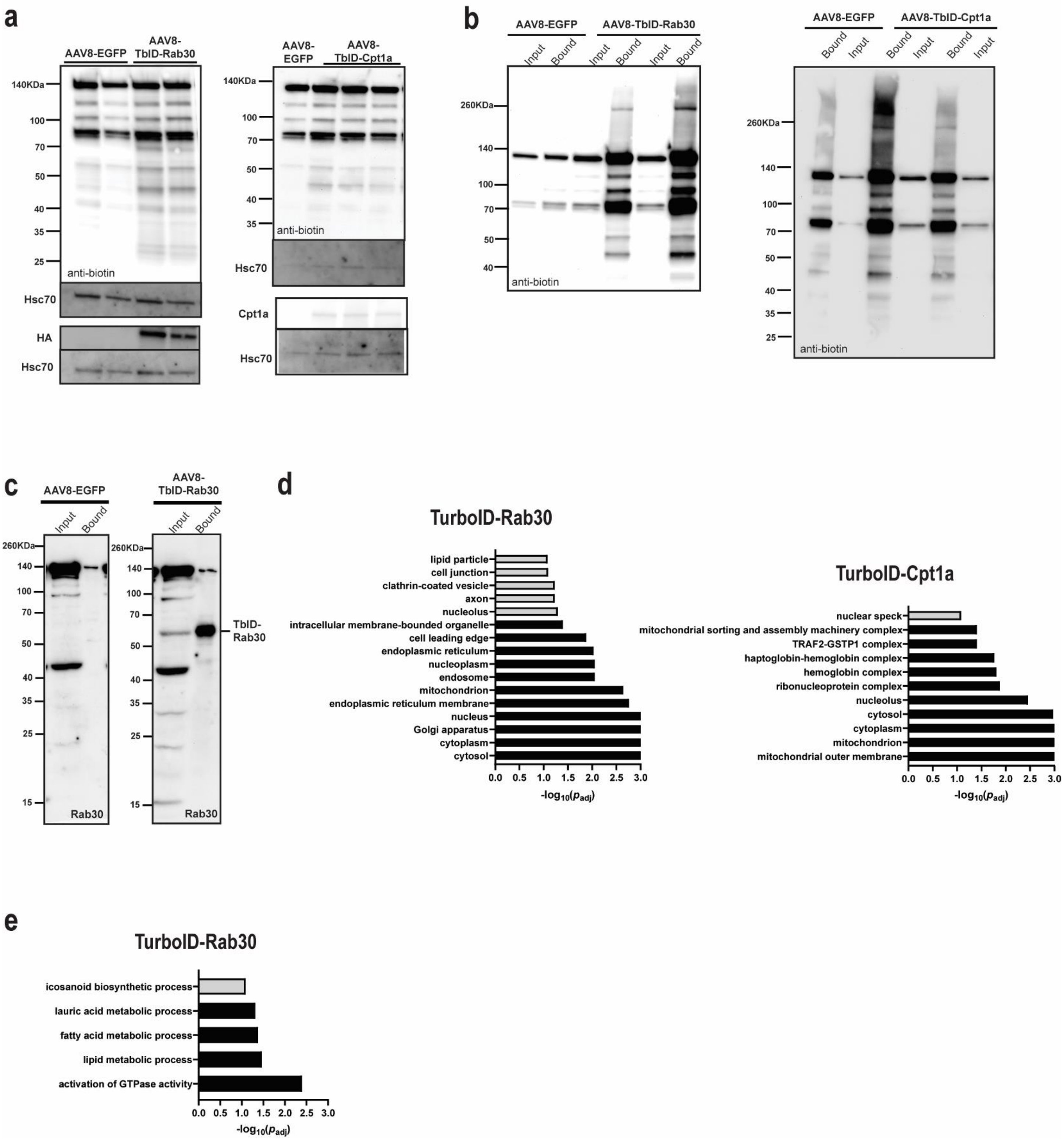
a
AAV8-EGFP
AAV8-TbID-Rab30
140KDa
100
70
50
40
35
25
anti-biotin
Hsc70
HA
Hsc70
AAV8-EGFP
AAV8-TbID-Cpt1a
140KDa
100
70
50
40
35
anti-biotin
Hsc70
Cpt1a
Hsc70
b
AAV8-EGFP
AAV8-TbID-Rab30
Input
Bound
Input
Bound
Input
Bound
260KDa
140
100
70
50
40
anti-biotin
AAV8-EGFP
AAV8-TbID-Cpt1a
Bound
Input
Bound
Input
Bound
Input
260KDa
140
100
70
50
40
35
25
anti-biotin
c
AAV8-EGFP
AAV8-TbID-Rab30
Input
Bound
Input
Bound
260KDa
140
100
70
50
40
35
25
15
Rab30
TbID-Rab30
Rab30
d
TurboID-Rab30
lipid particle
cell junction
clathrin-coated vesicle
axon
nucleolus
intracellular membrane-bounded organelle
cell leading edge
endoplasmic reticulum
nucleoplasm
endosome
mitochondrion
endoplasmic reticulum membrane
nucleus
Golgi apparatus
cytoplasm
cytosol
0.0 0.5 1.0 1.5 2.0 2.5 3.0
-log10(padj)
TurboID-Cpt1a
nuclear speck
mitochondrial sorting and assembly machinery complex
TRAF2-GSTP1 complex
haptoglobin-hemoglobin complex
hemoglobin complex
ribonucleoprotein complex
nucleolus
cytosol
cytoplasm
mitochondrion
mitochondrial outer membrane
0.0 0.5 1.0 1.5 2.0 2.5 3.0
-log10(padj)
e
TurboID-Rab30
icosanoid biosynthetic process
lauric acid metabolic process
fatty acid metabolic process
lipid metabolic process
activation of GTPase activity
0.0 0.5 1.0 1.5 2.0 2.5 3.0
-log10(padj)

Supplementary Fig. 2. TurboID technology to define the Rab30 interactome *in vivo.* **a** Representative immunoblot for total biotinylation (anti-biotin), HA-tag, and Cpt1a in 30μg liver homogenate from mice expressing AAV8-EGFP, AAV8-3xHA-TurboID-Rab30, or AAV8-TurboID-Cpt1a 3hr post biotin injection. Hsc70 is a total protein loading control. **b** Representative immunoblot following enrichment of biotinylated proteins on streptavidin beads from liver homogenate of biotin injected AAV8-EGFP, AAV8-3xHA-TurboID-Rab30, or AAV8-TurboID-Cpt1a expressing animals. "Input" lanes reflect ~10μg protein (4% of total loaded onto streptavidin beads), while "Bound" lanes (peptides eluted from the immunoprecipitation) reflect 40% of total eluted fraction. **c** Representative immunoblot for Rab30 following enrichment of biotinylated proteins on streptavidin beads from 1mg liver homogenate of biotin injected AAV8-EGFP or AAV8-3xHA-TurboID-Rab30 expressing animals. "Input" lanes reflect ~20μg protein, while "Bound" lanes (peptides eluted from the immunoprecipitation) reflect 2% of total immunoprecipitation. **d** Gene Ontology for cellular component (GO_CC) vs -log(p_{adj}) for proteins identified by TurboID-Rab30 and TurboID-Cpt1a in mouse livers. Pathway terms are ranked against the Benjamini-adjusted *p*-value generated by the DAVID functional annotation tool. GO terms with p_{adj}<0.05 are depicted in black and p_{adj}<0.1 are depicted in grey. **e** Gene Ontology for biological process (GO_BP) vs -log(p_{adj}) for proteins identified by TurboID-Rab30 in mouse livers. Pathway terms are ranked against the Benjamini-adjusted *p*-value generated by the DAVID functional annotation tool. GO terms with p_{adj}<0.05 are depicted in black and p_{adj}<0.1 are depicted in grey. Source data are provided as a Source Data file.

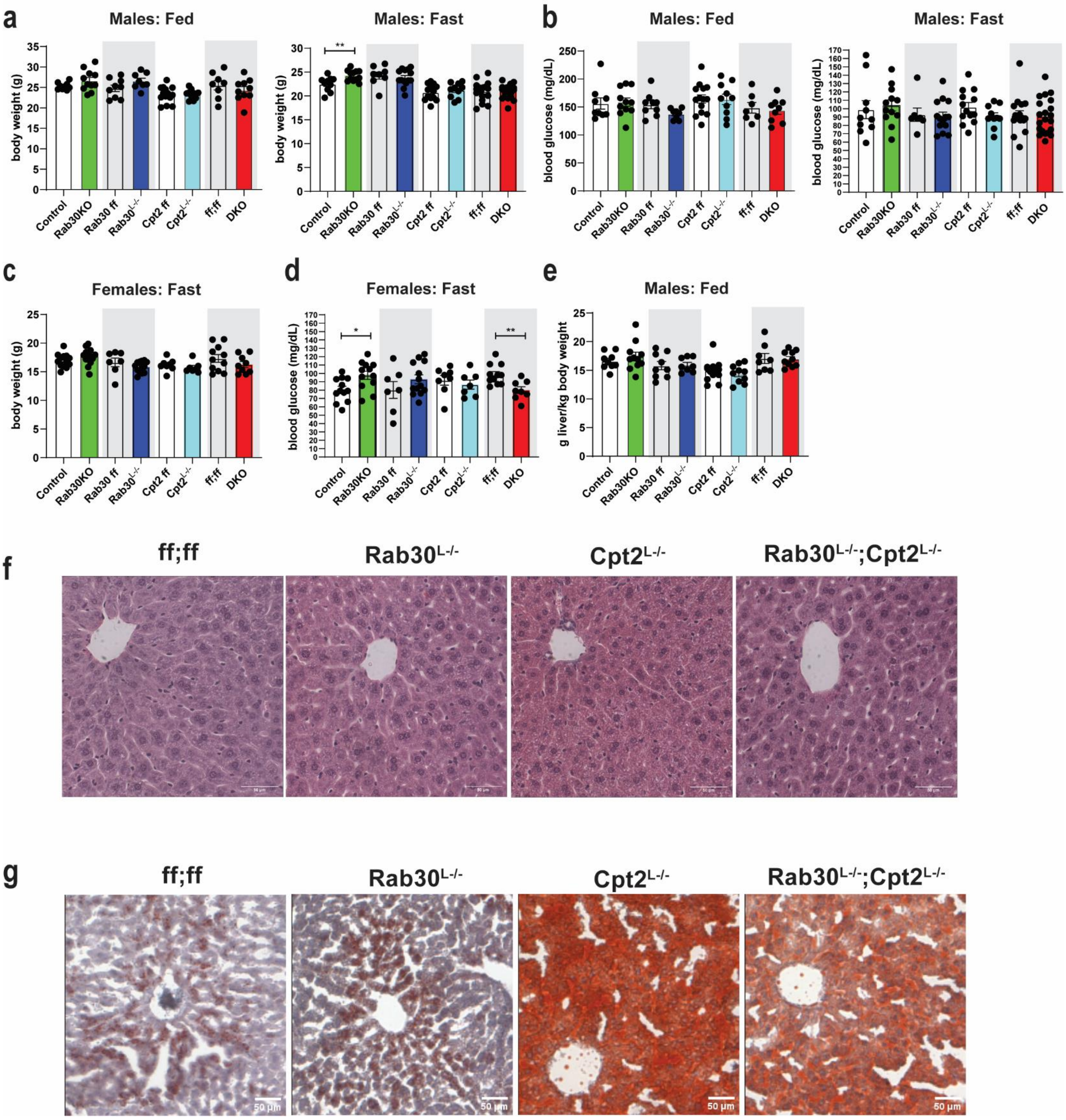

a
Males: Fed
body weight (g)
Males: Fast
b
Males: Fed
blood glucose (mg/dL)
Males: Fast
Control
Rab30KO
Rab30 ff
Rab30L-/-
Cpt2 ff
Cpt2L-/-
ff;ff
DKO
c
Females: Fast
d
Females: Fast
e
Males: Fed
g liver/kg body weight
f
ff;ff
Rab30L-/-
Cpt2L-/-
Rab30L-/-;Cpt2L-/-
g
ff;ff
Rab30L-/-
Cpt2L-/-
Rab30L-/-;Cpt2L-/-
50 µm

Supplementary Fig. 3. Fed and fasting state body weight, blood glucose, liver weights, and histology of Rab30 knockout models. a Fed state (2-4hr fasted) and 24hr fasted body weight of 9-11 week old male mice. **b** Fed state (2-4hr fasted) and 24hr fasted blood glucose of 9-11 week old male mice. **c** 24hr fasted body weight of 9-11 week old female mice. **d** 24hr fasted blood glucose of 9-11 week old female mice. **e** Wet liver weights (large left lobe) normalized to body weight of fed-state male mice. **f** Fed state histology of H&E stained livers from control, Rab30KO, $Cpt2^{L-/-}$, and Rab30;Cpt2 DKO male mice. Scale bar represents 50μm. **g** 24hr fasted Oil Red O stained livers from control, Rab30KO, $Cpt2^{L-/-}$, and Rab30;Cpt2 DKO male mice. Scale bar represents 50μm. Staining was repeated for 2 mice per genotype. For panels **a-e**, asterisks denote significance between knockouts and their littermate controls (Control, Rab30 ff, Cpt2 ff, and ff;ff for Rab30KO, $Rab30^{L-/-}$, $Cpt2^{L-/-}$, and DKO, respectively). Significance was determined by two-tailed *t*-test: *, $p<0.05$; **, $p<0.01$; ***, $p<0.001$, ****, $p<0.0001$. All n, *p*-values, and source data for relevant panels are provided in the Source Data file.

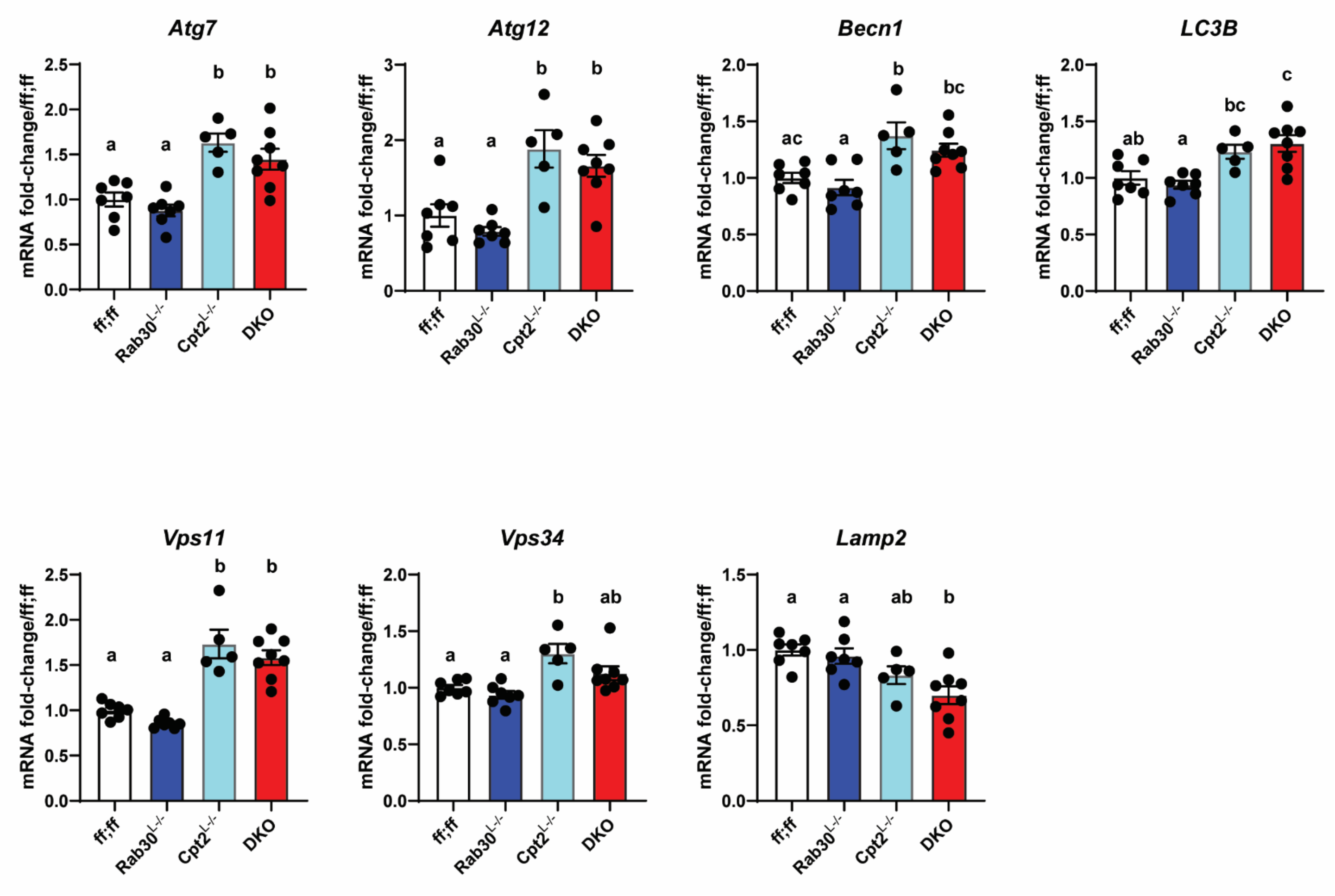

Atg7
Atg12
Becn1
LC3B
Vps11
Vps34
Lamp2
mRNA fold-change/ff;ff
ff;ff
Rab30L-/-
Cpt2L-/-
DKO
a
b
ab
ac
bc
c
0.0
0.5
1.0
1.5
2.0
2.5
0
1
2
3

Supplementary Fig. 4. Loss of Rab30 does not affect the mRNA of genes involved in autophagosome formation. qRT-PCR in the livers of 24hr fasted males (n=7 ff;ff and Rab30$^{L-/-}$, 5 Cpt2$^{L-/-}$, and 8 DKO). Values are mean±SEM relative to ff;ff. Letters indicate significance groups by Tukey's multiple comparisons test following one-way ANOVA. ANOVA tables and source data for relevant panels are provided as a Source Data file.

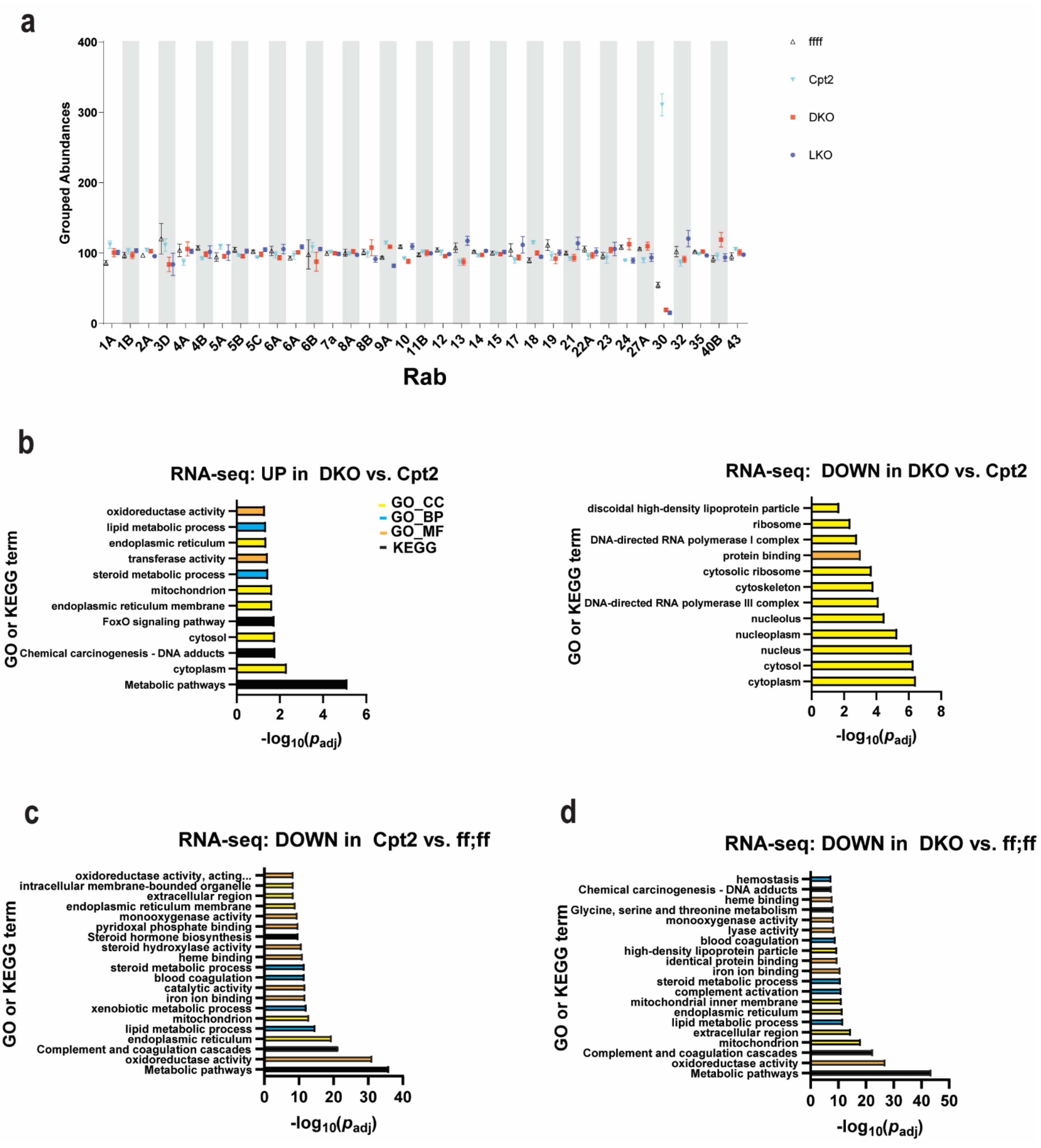
a
Grouped Abundances
400
300
200
100
0
1A 1B 2A 3D 4A 4B 5A 5B 5C 6A 6A 6B 7a 8A 8B 9A 10 11B 12 13 14 15 17 18 19 21 22A 23 24 27A 30 32 35 40B 43
Rab
ffff
Cpt2
DKO
LKO
b
RNA-seq: UP in DKO vs. Cpt2
GO_CC
GO_BP
GO_MF
KEGG
GO or KEGG term
oxidoreductase activity
lipid metabolic process
endoplasmic reticulum
transferase activity
steroid metabolic process
mitochondrion
endoplasmic reticulum membrane
FoxO signaling pathway
cytosol
Chemical carcinogenesis - DNA adducts
cytoplasm
Metabolic pathways
0 2 4 6
-log10(padj)
RNA-seq: DOWN in DKO vs. Cpt2
GO or KEGG term
discoidal high-density lipoprotein particle
ribosome
DNA-directed RNA polymerase I complex
protein binding
cytosolic ribosome
cytoskeleton
DNA-directed RNA polymerase III complex
nucleolus
nucleoplasm
nucleus
cytosol
cytoplasm
0 2 4 6 8
-log10(padj)
c
RNA-seq: DOWN in Cpt2 vs. ff;ff
GO or KEGG term
oxidoreductase activity, acting...
intracellular membrane-bounded organelle
extracellular region
endoplasmic reticulum membrane
monooxygenase activity
pyridoxal phosphate binding
Steroid hormone biosynthesis
steroid hydroxylase activity
heme binding
steroid metabolic process
blood coagulation
catalytic activity
iron ion binding
xenobiotic metabolic process
mitochondrion
lipid metabolic process
endoplasmic reticulum
Complement and coagulation cascades
oxidoreductase activity
Metabolic pathways
0 10 20 30 40
-log10(padj)
d
RNA-seq: DOWN in DKO vs. ff;ff
GO or KEGG term
hemostasis
Chemical carcinogenesis - DNA adducts
heme binding
Glycine, serine and threonine metabolism
monooxygenase activity
lyase activity
blood coagulation
high-density lipoprotein particle
identical protein binding
iron ion binding
steroid metabolic process
complement activation
mitochondrial inner membrane
endoplasmic reticulum
lipid metabolic process
extracellular region
mitochondrion
Complement and coagulation cascades
oxidoreductase activity
Metabolic pathways
0 10 20 30 40 50
-log10(padj)

Supplementary Fig. 5. Protein abundances of Rabs in fasted knockout livers and pathway analysis of RNA-seq data. **a** Grouped abundances of Rab family members detected in 24hr fasted proteomics (n=4/genotype). Data are represented as average ± SEM. **b** Gene ontology and KEGG pathway analysis of RNA-seq comparing up- and down-regulated pathways in the DKO vs. $Cpt2^{L-/-}$ fasted livers. **c** Gene ontology and KEGG pathway analysis of RNA-seq for downregulated pathways in the $Cpt2^{L-/-}$ vs. Rab30; Cpt2 floxed (ff;ff) control fasted livers. **d** Gene ontology and KEGG pathway analysis of RNA-seq for downregulated pathways in the DKO vs. Rab30; Cpt2 floxed (ff;ff) control fasted livers. Genes with fold-change of at least 1.2 and $p_{adj}<0.05$ were submitted for pathway analysis to the DAVID functional annotation tool. Pathway terms are ranked against the Benjamini-adjusted *p*-value generated by the DAVID functional annotation tool. Colors represent pathway class as denoted in the legend. Source data for relevant panels are provided as a Source Data file.

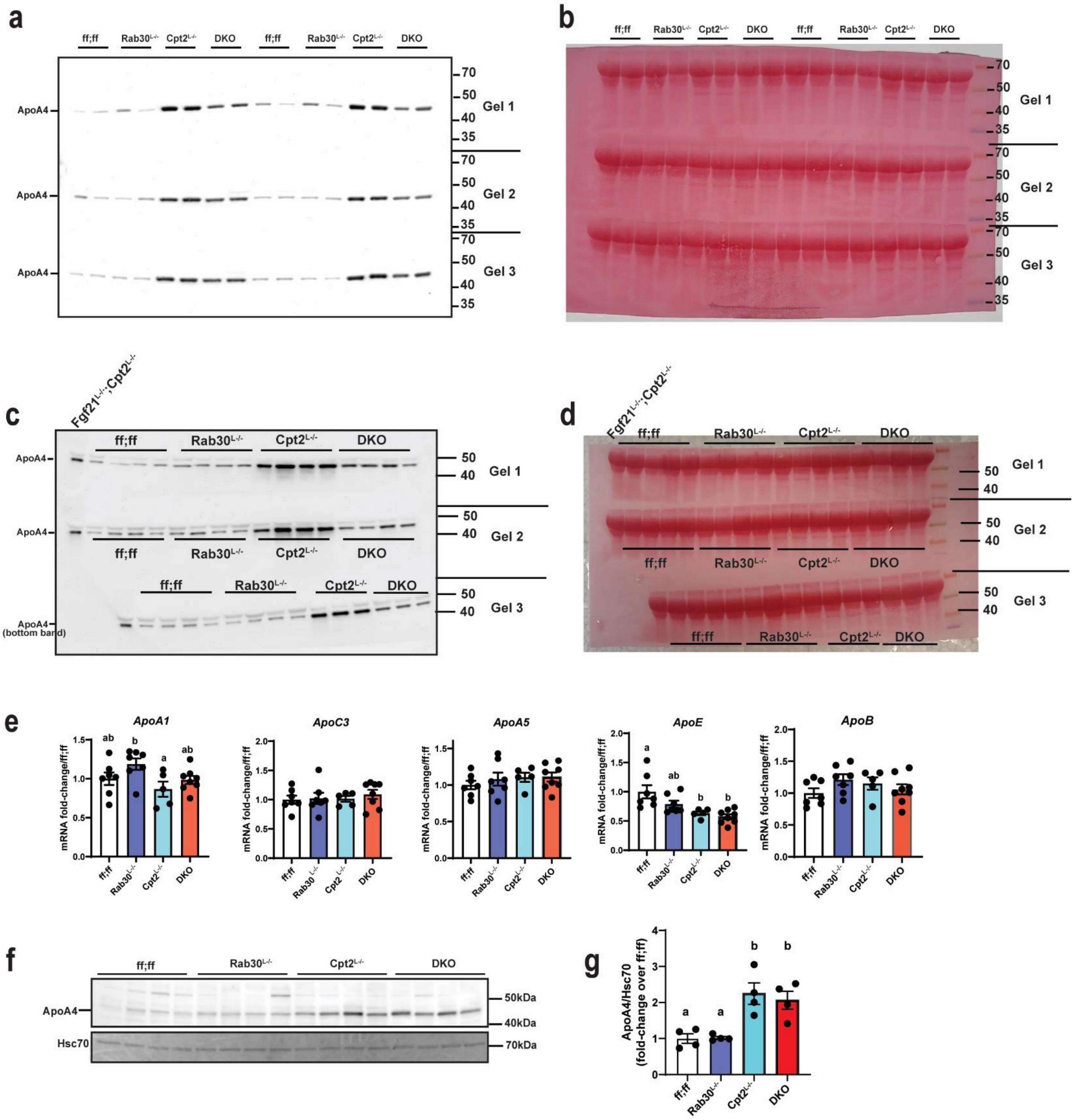
a
ff;ff
Rab30L-/-
Cpt2L-/-
DKO
ApoA4
Gel 1
Gel 2
Gel 3
70
50
40
35
b
c
Fgf21L-/-;Cpt2L-/-
ApoA4 (bottom band)
d
e
ApoA1
ApoC3
ApoA5
ApoE
ApoB
mRNA fold-change/ff;ff
ab
b
a
f
ApoA4
Hsc70
50kDa
40kDa
70kDa
g
ApoA4/Hsc70 (fold-change over ff;ff)

Supplementary Fig. 6. ApoA4 abundance in the serum and livers of fasted knockouts. a,b Immunoblot for ApoA4 (**a**) and Ponceau S protein stain (**b**) in the serum (1µL/lane) of 24hr fasted males. Proteins from n=12 serum samples/genotype were resolved on 3 separate 4-15% SDS-PAGE gels (4 samples/genotype per gel) prior to being transferred onto the same PVDF membrane and probed for the protein of interest. **c,d** Immunoblot for ApoA4 (**c**) and Ponceau S protein stain (**d**) in the serum (1µL/lane) of 24hr fasted females. Proteins from n=12 serum samples for ff;ff and Rab30$^{L-/-}$ and n=11 for Cpt2$^{L-/-}$ and DKO were resolved on 3 separate 4-15% SDS-PAGE gels (4 samples/genotype per gel) prior to being transferred onto the same PVDF membrane and probed for the protein of interest. Serum from an Fgf21$^{L-/-}$;Cpt2$^{L-/-}$ animal is in the first lane of each row. The last row (Gel 3) has 3 Cpt2$^{L-/-}$ and 3 DKO samples, while all other rows have 4 each. **e** qRT-PCR of select apolipoproteins in 24hr fasted male livers (n=7/genotype, except n=5 for Cpt2$^{L-/-}$). Letters indicate significance groups by Tukey's multiple comparisons test following one-way ANOVA. **f, g** ApoA4 immunoblot (**f)** and quantification (**g**) in the livers of 24hr fasted male mice (n=4/genotype). Hsc70 is an equal protein loading control. ApoA4 signal was normalized to Hsc70 signal and the quantification is represented as fold-change over ff;ff signal. Letters indicate significance groups by Tukey's multiple comparisons test following one-way ANOVA. ANOVA tables and source data for relevant panels are provided as a Source Data file.

Supplementary Table 1. List of qPCR primer sequences

Gene	Forward Primer Sequence	Reverse Primer Sequence
Rab30	GGTTGCGGGAGATAGAACAG	GCCTCTGAGAACTCTTCTGCT
Pdk4	ATCTAACATCGCCAGAATTAAACC	GGAACGTACACAATGTGGATTG
Cpt2	CAACTCGTATACCCAAACCCAGTC	GTTCCCATCTTGATCGAGGACATC
Becn1	GGAAAAGAACCGCAACGTGGTG	AAACTGTCCGCTGTGCCAGATG
Atg12	GGCCTCGGAACAGTTGTTTA	CAGCACCGAAATGTCTCTGA
Atg7	TGCCTATGATGATCTGTGTC	CACCAACTGTTATCTTTGTCC
Vps11	AAAAGAGAGACGGTGGCAATC	AGCCCAGTAACGGGATAGTTG
Vps34	CCTGGACATCAACGTGCAG	TGTCTCTTGGTATAGCCCAGAAA
LC3B	CCCACCAAGATCCCAGTGAT	CCAGGAACTTGGTCTTCTCCA
Lamp2	GATGTGCCTCTCTCCGGTTA	ATTGGACTGAACGGCTCCTA
Fgf21	CTGCTGGGGGTCTACCAAG	CTGCGCCTACCACTGTTCC
ApoC3	GAGGGATCCTTGCTGCTGG	AGTGCATCCTGGACCGTCTT
ApoAI	AGAAGAGCTGGACACCCAGA	CACCACAGCTTTCATCCTGA
ApoA4	CCAGCTAAGCAACAATGCC	TGGAAGAGGGTACTGAGCTGC
ApoA5	GACACCTACCTGCAGATTGCT	TTCCCACAGGTCGTCCAGTCG
ApoE	CTCCCAAGTCACACAAGAACT	CCAGCTCCTTTTTGTAAGCCTTT
ApoB	AAGCACCTCCGAAAGTACGTG	CTCCAGCTCTACCTTACAGTTGA
CycloA (Ppia)	AGCACTGGGGAGAAAGGATT	CATGCCTTCTTTCACCTTCC
18S (Rn18s-rs5)	GCAATTATTCCCCATGAACG	GGCCTCACTAAACCATCCAA
B-actin	GGCTGTATTCCCCTCCATCG	CCAGTTGGTAACAATGCCATGT
Srebp1c	GGAGCCATGGATTGCACATT	GGCCCGGGAAGTCACTGT
Acc1	TGTACAAGCAGTGTGGGCTGGCT	CCACATGGCCTGGCTTGGAGGG
Scd1	CCCTGCGGATCTTCCTTATC	TGTGTTTCTGAGAACTTGTGGTG
Fasn	GCTGCGGAAACTTCAGGAAAT	AGAGACGTGTCACTCCTGGACTT

Supplementary Table 2. List of antibodies

Antigen	Supplier	Catalog #	Species	Use	Dilution
Rab30	LSBio	LS-C353400	rabbit	WB	1:500-1:1000
Cpt1a	Proteintech	15184-1-AP	rabbit	WB	1:500-1:1000
Hsc70	SC	7298	mouse	WB	1:1000
GM130	abcam	ab52649	rabbit	IHC	1:250
ApoA4	R&D Systems	AF8125	sheep	WB	1:1000
Beclin-1	Cell Signaling	3495T	rabbit	WB	1:1000
LC3A/B	Cell Signaling	12741T	rabbit	WB	1:1000
HA	Sigma	H6908	rabbit	WB	1:1000
rabbit IgG (Alexa Fluor 488)	Invitrogen	A11008	goat	IHC	1:500
rabbit IgG (HRP-linked)	Cell Signaling	7074S	goat	WB	1:5000-10,000
anti-mouse IgG (Cy3 conjugate)	Invitrogen	M30010	goat	WB	1:2000
anti-sheep (HRP-linked)	Sigma	A3415	donkey	WB	1:5000
