# Supplementary material for: Rab30 facilitates lipid homeostasis during fasting
Source: Nat Commun. 2024 May 25;15:4469. doi: 10.1038/s41467-024-48959-x (PMC11127972; doi:10.1038/s41467-024-48959-x)
Supplement: Supplementary file 3 — Description of Additional Supplementary Files [file 41467_2024_48959_MOESM3_ESM.pdf]

File Name: Supplementary Data 1

Description: Proteins identified by yeast two-hybrid of Rab30Q68L(1-198aa) against human liver cDNA library and associated Gene Ontology Cellular Component (GO_CC) terms

File Name: Supplementary Data 2

Description: Proteins identified in replicates of TurboID-Rab30 or TurboID-Cpt1a livers post-normalization and filtering

File Name: Supplementary Data 3

Description: Total list of proteins identified across all replicates of TurboID-Rab30 and TurboID-Cpt1a streptavidin pulldowns prior to normalizing and filtering

File Name: Supplementary Movie 1

Description: HA-mScarletI-Rab30 membrane protraction event and vesicle dynamics in primary mouse hepatocytes. Time lapse of AAV8-mScarletI-Rab30 membrane protraction event in primary mouse hepatocytes corresponding to the still images depicted in Fig. 2E. 60x oil objective, zoomed in.

File Name: Supplementary Movie 2

Description: HA-mScarletI-Rab30 and C6-NBD-Ceramide Golgi stain dynamics in live primary mouse hepatocytes. Time lapse (~4x speed) of AAV8-mScarletI-Rab30 in primary mouse hepatocytes stained with the live-cell Golgi marker C6-NBD-Ceramide and nuclear stain Hoescht 33342 (channel not acquired during imaging). 60x oil objective.

File Name: Supplementary Movie 3

Description: HA-mScarletI-Rab30 and C6-NBD-Ceramide Golgi stain tubular networks in live primary mouse hepatocytes. Time lapse (~4x speed) of AAV8-mScarletI-Rab30 in primary mouse hepatocytes stained with the live-cell Golgi marker C6-NBD-Ceramide and nuclear stain Hoescht 33342. 60x oil objective.
