# Supplementary material for: Rab30 facilitates lipid homeostasis during fasting
Source: Nat Commun. 2024 May 25;15:4469. doi: 10.1038/s41467-024-48959-x (PMC11127972; doi:10.1038/s41467-024-48959-x)
Supplement: Supplementary file 10 — Reporting Summary [file 41467_2024_48959_MOESM10_ESM.pdf]

nature portfolio

Corresponding author(s): Michael Wolfgang

Last updated by author(s): 5/4/2024

Reporting Summary

Nature Portfolio wishes to improve the reproducibility of the work that we publish. This form provides structure for consistency and transparency in reporting. For further information on Nature Portfolio policies, see our Editorial Policies and the Editorial Policy Checklist.

Statistics

For all statistical analyses, confirm that the following items are present in the figure legend, table legend, main text, or Methods section.

n/a	Confirmed	
☐	☒	The exact sample size (*n*) for each experimental group/condition, given as a discrete number and unit of measurement
☐	☒	A statement on whether measurements were taken from distinct samples or whether the same sample was measured repeatedly
☐	☒	The statistical test(s) used AND whether they are one- or two-sided<br>*Only common tests should be described solely by name; describe more complex techniques in the Methods section.*
☐	☒	A description of all covariates tested
☐	☒	A description of any assumptions or corrections, such as tests of normality and adjustment for multiple comparisons
☐	☒	A full description of the statistical parameters including central tendency (e.g. means) or other basic estimates (e.g. regression coefficient) AND variation (e.g. standard deviation) or associated estimates of uncertainty (e.g. confidence intervals)
☐	☒	For null hypothesis testing, the test statistic (e.g. *F*, *t*, *r*) with confidence intervals, effect sizes, degrees of freedom and *P* value noted<br>*Give P values as exact values whenever suitable.*
☒	☐	For Bayesian analysis, information on the choice of priors and Markov chain Monte Carlo settings
☒	☐	For hierarchical and complex designs, identification of the appropriate level for tests and full reporting of outcomes
☒	☐	Estimates of effect sizes (e.g. Cohen's *d*, Pearson's *r*), indicating how they were calculated

Our web collection on statistics for biologists contains articles on many of the points above.

Software and code

Policy information about availability of computer code

Data collection	Confocal microscopy images were collected with Zen (Zeiss) 2.3 SP1; RNA sequencing data was collected remotely by Novogene Corporation Inc.; Proteomics data was collected by the Johns Hopkins School of Medicine Mass Spectrometry and Proteomics Facility; Western blot images were acquired using the AlphaInnotech software AlphaView 1.3.0.6; qRT-PCR data were collected with the BioRad CFX Manager 2.1; Absorbance readings for serum biochemistry and liver triglycerides were collected with Gen5 1.09 and 3.13 softwares
Data analysis	GraphPad Prism 8.1.2 (332); Fiji 2.3.051; NIS-Elements Viewer 4.50; R version 4.1.3 DESeq2 3.17; AlphaView 1.3.0.6; Proteome Discoverer version 2.4.0.305; Mascot 2.8

For manuscripts utilizing custom algorithms or software that are central to the research but not yet described in published literature, software must be made available to editors and reviewers. We strongly encourage code deposition in a community repository (e.g. GitHub). See the Nature Portfolio guidelines for submitting code & software for further information.

Data

Policy information about availability of data

All manuscripts must include a data availability statement. This statement should provide the following information, where applicable:

- Accession codes, unique identifiers, or web links for publicly available datasets
- A description of any restrictions on data availability
- For clinical datasets or third party data, please ensure that the statement adheres to our policy

RNA-seq data were deposited in GSE240396 [https://www.ncbi.nlm.nih.gov/geo/query/acc.cgi?acc=GSE240396]. The mass spectrometry proteomics data have been deposited to the ProteomeXchange Consortium via the PRIDE partner repository with the dataset identifier PXD044528 [https://proteomecentral.proteomexchange.org/cgi/GetDataset?ID=PXD044528] for whole liver proteome and PXD050376 [https://www.ebi.ac.uk/pride/archive/projects/PXD050376], for the proteins identified from TurboID following streptavidin pulldown. Source data are provided with this paper.

Research involving human participants, their data, or biological material

Policy information about studies with human participants or human data. See also policy information about sex, gender (identity/presentation), and sexual orientation and race, ethnicity and racism.

Reporting on sex and gender	*Use the terms sex (biological attribute) and gender (shaped by social and cultural circumstances) carefully in order to avoid confusing both terms. Indicate if findings apply to only one sex or gender; describe whether sex and gender were considered in study design; whether sex and/or gender was determined based on self-reporting or assigned and methods used. Provide in the source data disaggregated sex and gender data, where this information has been collected, and if consent has been obtained for sharing of individual-level data; provide overall numbers in this Reporting Summary. Please state if this information has not been collected. Report sex- and gender-based analyses where performed, justify reasons for lack of sex- and gender-based analysis.*
Reporting on race, ethnicity, or other socially relevant groupings	*Please specify the socially constructed or socially relevant categorization variable(s) used in your manuscript and explain why they were used. Please note that such variables should not be used as proxies for other socially constructed/relevant variables (for example, race or ethnicity should not be used as a proxy for socioeconomic status). Provide clear definitions of the relevant terms used, how they were provided (by the participants/respondents, the researchers, or third parties), and the method(s) used to classify people into the different categories (e.g. self-report, census or administrative data, social media data, etc.) Please provide details about how you controlled for confounding variables in your analyses.*
Population characteristics	*Describe the covariate-relevant population characteristics of the human research participants (e.g. age, genotypic information, past and current diagnosis and treatment categories). If you filled out the behavioural & social sciences study design questions and have nothing to add here, write "See above."*
Recruitment	*Describe how participants were recruited. Outline any potential self-selection bias or other biases that may be present and how these are likely to impact results.*
Ethics oversight	*Identify the organization(s) that approved the study protocol.*

Note that full information on the approval of the study protocol must also be provided in the manuscript.

Field-specific reporting

Please select the one below that is the best fit for your research. If you are not sure, read the appropriate sections before making your selection.

☒ Life sciences ☐ Behavioural & social sciences ☐ Ecological, evolutionary & environmental sciences

For a reference copy of the document with all sections, see nature.com/documents/nr-reporting-summary-flat.pdf

Life sciences study design

All studies must disclose on these points even when the disclosure is negative.

Sample size	Sample sizes used are consistent with similar studies in this field.
Data exclusions	None of the data presented on bar graphs were excluded from analyses. For pathway analysis of RNA sequencing and proteomics data, only genes and proteins in pairwise comparisons that were changed by at least 1.2-fold and had a p-adj value <0.05 were considered
Replication	Body weights, tissue weights, serum and liver biochemistry, and gene expression by qRT-PCR were tested once with a minimum of 5 animals. Western blots used 2-12 animals/genotype and/or condition as described in the text. Tissue histology, electron microscopy, and liver confocal microscopy images were performed on tissues obtained from at least 2 mice of the same age and sex. In vitro studies were performed in replicate wells/plates. All attempts to repeat the experiment were successful.
Randomization	Experimental mice were allocated based on age, sex, and genotype. Littermates were used when possible, and experiments were performed

Randomization	with mice across different litters and parents.
Blinding	Blinding was not performed in this study. Sex, age, and genotype. Littermates were used when possible, and experiments were performed with mice across different litters and parents.

Reporting for specific materials, systems and methods

We require information from authors about some types of materials, experimental systems and methods used in many studies. Here, indicate whether each material, system or method listed is relevant to your study. If you are not sure if a list item applies to your research, read the appropriate section before selecting a response.

Materials & experimental systems

n/a	Involved in the study
☐	☒ Antibodies
☐	☒ Eukaryotic cell lines
☒	☐ Palaeontology and archaeology
☐	☒ Animals and other organisms
☒	☐ Clinical data
☒	☐ Dual use research of concern
☒	☐ Plants

Methods

n/a	Involved in the study
☒	☐ ChIP-seq
☒	☐ Flow cytometry
☒	☐ MRI-based neuroimaging

Antibodies

Antibodies used

Rab30 LSBio LS C353400 Rabbit Lot#176184 (now LS�C808247) 1:500-1:1000
Hsc70 Santa Cruz 7298 mouse Lot#D2121 Clone B-6 1:1000
GM130 abcam ab52649 rabbit A-4 ClonEP892Y 1:250
ApoA4 R&D Systems AF8125 sheep CIGO011802A 1:1000
Beclin-1 Cell Signaling 3485T rabbit lot#2 Clone D40C5 1:1000
LC3A/B Cell Signaling 12741T rabbit Lot#3 Clone D304C XP(R) 1:1000
HA Sigma H6908 rabbit lot#110m4850 1:1000
rabbit IgG (Alexa Fluor488) Invitrogen A11008 goat lot# 2284594 1:500
rabbit IgG (HRP-linked) Cell Signaling 7074S goat lot#'s 28,29 1:5000-10,000
anti-mouse IgG (Cy3 conjugate) Invitrogen M30010 goat 2550967 1:2000
anti-sheep (HRP-linked) Sigma A3415 donkey 1:5000

Validation

https://www.lsbio.com/antibodies/rab30-antibody-if-immunofluorescence-wb-western-ls-c808247/834812
https://www.scbt.com/p/hsc-70-antibody-b-6
https://www.abcam.com/products/primary-antibodies/gm130-antibody-ep892y-cis-golgi-marker-ab52649.html
https://www.rndsystems.com/products/human-mouse-apolipoprotein-a-iv-apoa4-antibody_af8125
https://www.cellsignal.com/products/primary-antibodies/beclin-1-d40c5-rabbit-mab/3495
https://www.cellsignal.com/products/primary-antibodies/lc3a-b-d3u4c-xp-rabbit-mab/12741
https://www.sigmaaldrich.com/US/en/product/sigma/h6908
https://www.thermofisher.com/antibody/product/Goat-anti-Rabbit-IgG-H-L-Cross-Adsorbed-Secondary-Antibody-Polyclonal/A-11008
https://www.cellsignal.com/products/secondary-antibodies/anti-rabbit-igg-hrp-linked-antibody/7074
https://www.thermofisher.com/antibody/product/Goat-anti-Mouse-IgG-H-L-Cross-Adsorbed-Secondary-Antibody-Polyclonal/M30010
https://www.sigmaaldrich.com/US/en/product/sigma/a3415

Eukaryotic cell lines

Policy information about cell lines and Sex and Gender in Research

Cell line source(s)	Primary mouse hepatocytes (both male and female mice were used)<br>alpha mouse liver 12 (AML12)
Authentication	AML12 cells were purchased from ATCC:https://www.atcc.org/products/crl-2254
Mycoplasma contamination	Cell lines were not tested for mycoplasma contamination
Commonly misidentified lines<br>(See ICLAC register)	No commonly misidentified lines were used in this study and the cell lines were not authenticated

Animals and other research organisms

Policy information about studies involving animals; ARRIVE guidelines recommended for reporting animal research, and Sex and Gender in Research

Laboratory animals	Species: Mus musculus<br>Strain: C57/BL6J<br>Age: 5-7 weeks for TEM images; 8-10 weeks for all other experiments unless less otherwise stated in the Methods section of the Study (i.e., Atgl LKO animals injected with AAV8-TBG-mScarletI Rab30 were 14weeks old)
Wild animals	The study did not involve wild animals.
Reporting on sex	Described in the figure legends in detail. RNA sequencing, whole-liver proteomics, serum biochemistry, and liver lipid quantification were performed in male mice. Female mice were used for immunohistochemistry. General physiological assessments (ex., fed/fasting body weight, liver weight, blood glucose), primary hepatocyte isolation, western blots and ApoA4 quantification in Figure 7 were performed in male and female mice.
Field-collected samples	The study did not involve samples that were collected from the field.
Ethics oversight	All procedures were performed in accordance with the NIH's Guide for the Care and Use of Laboratory Animals and under the approval of the Johns Hopkins School of Medicine Animal Care and Use Committee.

Note that full information on the approval of the study protocol must also be provided in the manuscript.

Plants

Seed stocks	*Report on the source of all seed stocks or other plant material used. If applicable, state the seed stock centre and catalogue number. If plant specimens were collected from the field, describe the collection location, date and sampling procedures.*
Novel plant genotypes	*Describe the methods by which all novel plant genotypes were produced. This includes those generated by transgenic approaches, gene editing, chemical/radiation-based mutagenesis and hybridization. For transgenic lines, describe the transformation method, the number of independent lines analyzed and the generation upon which experiments were performed. For gene-edited lines, describe the editor used, the endogenous sequence targeted for editing, the targeting guide RNA sequence (if applicable) and how the editor was applied.*
Authentication	*Describe any authentication procedures for each seed stock used or novel genotype generated. Describe any experiments used to assess the effect of a mutation and, where applicable, how potential secondary effects (e.g. second site T-DNA insertions, mosiacism, off-target gene editing) were examined.*
